# Organotypic Culture of Physiologically Functional Adult Mammalian Retinas

**DOI:** 10.1371/journal.pone.0000221

**Published:** 2007-02-21

**Authors:** Amane Koizumi, Günther Zeck, Yixin Ben, Richard H. Masland, Tatjana C. Jakobs

**Affiliations:** 1 Massachusetts General Hospital, Harvard Medical School, Boston, Massachusetts, United States of America; 2 Systems and Computational Biology, Max Planck Institute of Neurobiology, Martinsried, Germany; 3 Burke Institute, White Plains, New York, United States of America; University of Washington, United States of America

## Abstract

**Background:**

The adult mammalian retina is an important model in research on the central nervous system. Many experiments require the combined use of genetic manipulation, imaging, and electrophysiological recording, which make it desirable to use an in vitro preparation. Unfortunately, the tissue culture of the adult mammalian retina is difficult, mainly because of the high energy consumption of photoreceptors.

**Methods and Findings:**

We describe an interphase culture system for adult mammalian retina that allows for the expression of genes delivered to retinal neurons by particle-mediated transfer. The retinas retain their morphology and function for up to six days— long enough for the expression of many genes of interest—so that effects upon responses to light and receptive fields could be measured by patch recording or multielectrode array recording. We show that a variety of genes encoding pre- and post-synaptic marker proteins are localized correctly in ganglion and amacrine cells.

**Conclusions:**

In this system the effects on neuronal function of one or several introduced exogenous genes can be studied within intact neural circuitry of adult mammalian retina. This system is flexible enough to be compatible with genetic manipulation, imaging, cell transfection, pharmacological assay, and electrophysiological recordings.

## Introduction

Several features make the retina an appealing model of the mammalian central nervous system. It is the most accessible part of the CNS, and its thin, layered structure can be regarded as a stack of two dimensional arrays. The flow of information is essentially unidirectional toward the ganglion cells, the only projection neurons in the system. Most of the diverse functional cell types in the mammalian retina have been identified at least morphologically, and in some cases also physiologically [1], [2].

An approach to this highly complex system would be to selectively alter the function of identified neurons within the functional neural network. It would in principle be possible to create transgenic mice to perform such studies. An alternative is to inject viral vectors carrying the gene of interest into the eye. Though these are well-established technologies, drawbacks are relatively high cost and the long time required for each experiment. Here we sought to develop a more flexible and quickly responsive system.

The system combines features used for short-term recording from the retina with those used for longer term maintenance of brain slices. Retinas are difficult to culture, mainly because of the exceptionally high metabolism of the photoreceptors [3]. Yet, preparations for recording from rabbit retinas *in vitro* have been established that can be kept for over fifty hours [4]. These methods rely on constant superfusion of the tissue with oxygenated medium, and therefore require constant monitoring. On the other hand, brain slices in interphase chambers can be successfully maintained and manipulated in vitro for weeks [5]–[7]. Limited survival of retinas under relatively simple culture conditions has been demonstrated [8]–[13], most notably in elegant developmental studies by Wong and co-workers [14]. However, most of these experiments used neonatal or very young retinas, which are immature in most mammalian species and, because the photoreceptors are not functional, have low metabolic demands. In addition, most studies have been limited to incubations lasting less than 24 h. In order to provide a platform for experiments on the microcircuitry of the mature adult retina, we have established a hybrid interphase/perfusion system. We show that it is possible to record responses to light from rabbit retinas for up to six days in culture. This time is sufficient to allow for the expression of genes that have been introduced into individual retinal neurons. We have expressed a wide spectrum of proteins in retinas incubated in this way. As an illustration, we show here that fusion proteins of synaptic markers with GFP can be expressed within this time frame and appropriately localize within the dendritic tree.

## Results

### An organotypic interphase/perfusion culture of adult rabbit retina

**Figure 1:**
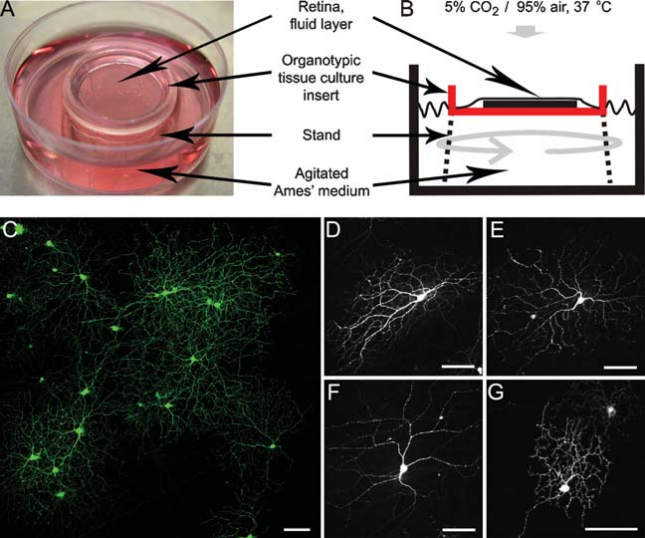
Incubation of rabbit retina. Photograph (**A**) and schematic diagram (**B**) of the hybrid interphase/perfusion chamber. Deep dishes are used with custom made “stands” of 1 cm height to support the tissue culture insert with the flat-mounted retina. The retina is in contact with the Ames' medium over the filter on the photoreceptor side; the ganglion cell side faces the atmosphere. (**C**) Low power micrograph of adult rabbit retina transfected with an expression plasmid for EGFP after 4 days in culture. Scale bar, 100 µm. (**D**) ON/OFF Directionally selective ganglion cell (Type G7, bistratified). All panels show maximum intensity projections of ∼20 individual image planes of a through-focus series taken at 1 µm steps on a confocal microscope. (**E**) ON directionally selective ganglion cell (G10). (**F**) Alpha cell. (**G**) Brisk sustained cell (G4). All scale bars, 100 µm.

Rabbit retinas were isolated and mounted ganglion cell side up on Millicell tissue culture filters (Figure 1). Directly afterwards the retinas were transfected via particle-mediated transfer of expression plasmids and placed in a culture dish containing Ames' medium for up to six days. We experimented with two different designs of culture chambers: individual small dishes placed on a rocking platform and a large reservoir tank where the filters bearing the retinas were kept afloat in a custom made “boats”, and the medium was agitated using a magnetic stirrer. In both cases, we found that agitating the medium and exchanging it daily were necessary to keep the retinas in good condition. The first design, mechanically similar to an ordinary tissue culture dish, was easier to use.

**Figure 2:**
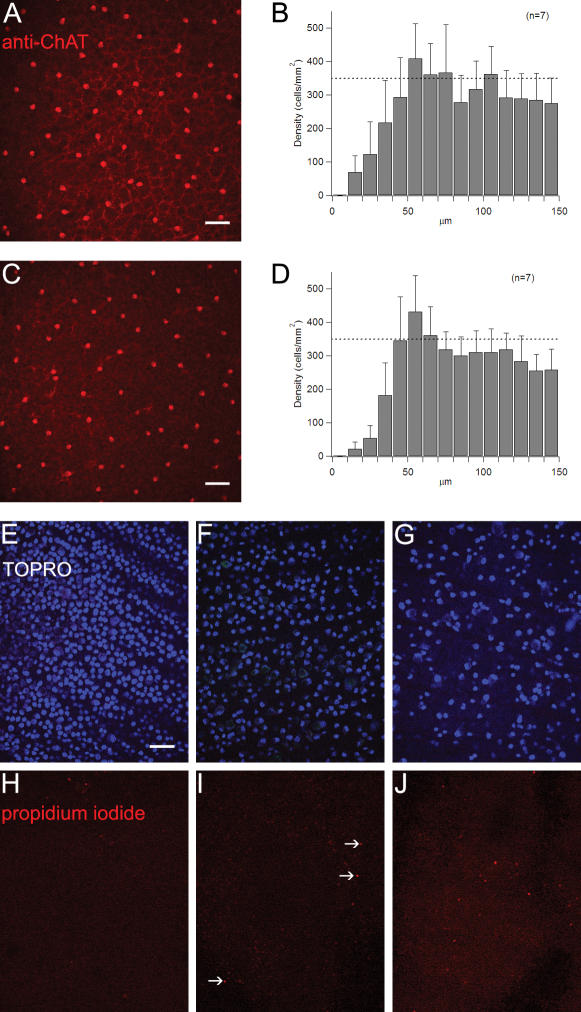
Viability of rabbit retina after six days of incubation. (**A**), Typical piece of rabbit retina stained with an antibody against ChAT (red) six after six days of incubation (focus on the ganglion cell layer). Seven such fields were measured to arrive at a density recovery profile (**B**) for starburst amacrine cells. Scale bar for A & C, 50 µm. (**C**), matched field with focus on the inner nuclear layer (OFF starburst amacrine cells). (**D**), density recovery profile for starburst amacrine cells in the INL. (**E, F, and G**), ganglion cell layer of rabbit retina incubated for six days stained for dead cells (propidium iodide, red), cells in the process of apoptosis (YO-PRO, bright green) and counterstained with the nuclear dye TOPRO (blue). No dead or dying cells are visible in the ganglion cell layer in central retina (panel E), mid-periphery (panel F), and peripheral retina (panel G). (**H, I, and J**) show matched fields with the focus on the photoreceptor cell bodies. Several cells are labeled by their incorporation of propidium iodide (arrows), indicating cell death in the photoreceptor layer. Scale bar for panels E–J, 50 µm.

Two to six days post transfection the tissue was evaluated. We never observed any gross abnormalities of the retinas in vertical sections (Figure S1 & S2). Individual GFP-labeled ganglion and amacrine cells were compared to the well-established “catalog” of neuron morphology in the rabbit retina [15], [16]. We observed specimens of all types of ganglion cells. Though we did observe some axonal retraction and occasional swollen dendrites, the ganglion cells showed no major abnormalities (see Figure 1 C–G). As a test for viability of amacrine cells, we stained the retinas with an antibody against choline acetyltransferase (ChAT) 6 days after transfection. Density recovery profile (DRP) analysis [17], [18] verified that the ChAT+ amacrine cells had the characteristic mosaic distribution after incubation (Figure 2 A–D). This indicates that the cells retained their normal spatial arrangement and there was no detectable dropout of cells of this type. In addition, the retinas were stained for degenerating and apoptotic cells. After 6 days, some cell death was beginning to be evident for all cell types (Figure 2 E–J), but it was numerically slight. Cell death in the ganglion cell layer, where the cells are readily quantified, was less than <0.5% of the total neuron present.

### Electrophysiological recordings from ganglion cells

**Figure 3:**
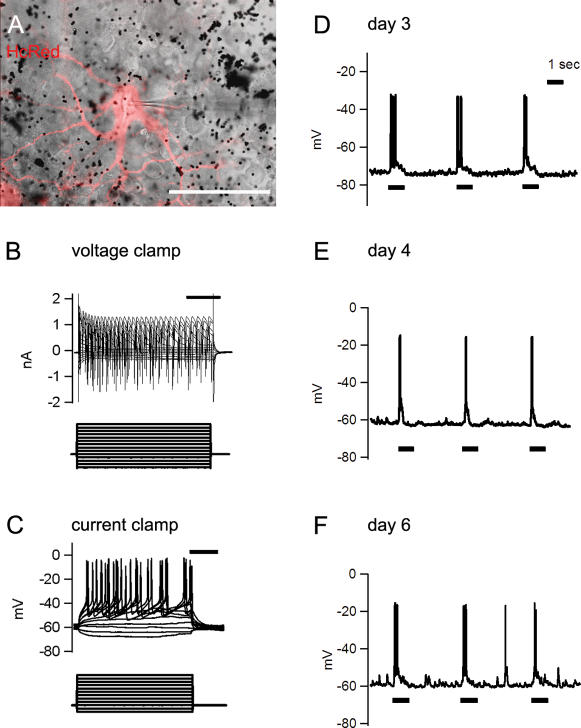
Patch-clamp recordings and light responses from ganglion cells in the incubated retina. (**A**) HcRed-transfected ON-type retinal ganglion cells after 3 day incubation. The patch-clamp pipette is seen (its light responses shown in C). Black dots are unincorporated gold particles. Scale bar, 50 µm. (**B**) An example of voltage clamp and current clamp recordings after 6 day incubation. All of recorded ganglion cells (n = 30) generated action potentials in response to current injection under current clamp configuration. In the voltage clamp, the cells were voltage-clamped at −77 mV and then command pulses were applied from −117 mV to 13 mV, in 10 mV steps, for 200 msec. In the current clamp, command pulses were applied from −40 pA to +180 pA, in 20 pA steps, for 200 msec. Scale bars are 50 msec. (**C–E**) light responses of ganglion cells from 3 day (the cell shown in A), 4 day, and 6 day-incubated retina, respectively, recorded under current clamp.

The ganglion cells had many normal properties in functional assays. Whole-cell patch-clamp recordings from 30 ganglion cells in retinas incubated for up to 6 days showed that the mean resting membrane potential was −70±9 mV (mean±SD), the normal range for retinal ganglion cells. All cells, including those after 6 days in culture (n = 6), generated normal action potentials when responding to current injection under current clamp configuration. The rate of firing increased in response to progressively more depolarizing current steps (Figure 3 B).

Retinas isolated under dim light and maintained in the dark for up to 4 days showed unequivocal responses to light in patch-clamp recordings (n = 10; Figure 3 C&D). After six days in culture, recording of light responses became unreliable, and was successful in only 3/6 cells tested (Figure 3 E).

**Figure 4:**
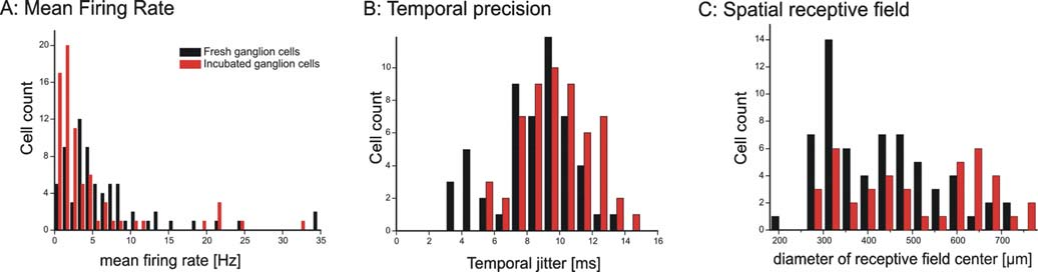
Electrophysiological properties of ganglion cells in incubated retinas are comparable to properties of freshly prepared ganglion cells. (**A**) Histogram of average firing rates for 69 ganglion cells from 5 retinas after 2 days (3 retinas) or 3 days (2 retinas) of incubation (red columns) and 73 ganglion cells from freshly prepared retinas (black columns). All cells were recorded with multielectrode arrays; the firing rate was calculated in response to spatial white noise (see Methods). The two distributions are similar, although more incubated cells are recorded with mean firing rates below 2 Hz. (**B**) Histogram of temporal jitter in response to the same spatially uniform temporal white noise stimulus. The temporal jitter distribution does not differ significantly (p<0.05, ANOVA) for the two populations. Mean temporal jitter was 10 milliseconds for incubated ganglion cells and 8.6 milliseconds for fresh ganglion cells. (**C**) The diameters of receptive field centers. Mean receptive field center was 520 µm for incubated ganglion cells (n = 43) and 430 µm for fresh ganglion cells (n = 73).

We examined the functional integrity of ganglion cell populations in incubated retinas using extracellular multielectrode recording. The mean firing rates of 69 ganglion cells from incubated retinas were compared to the activity of 73 ganglion cells from three fresh retinas (Fig. 4 A). The firing rates were calculated over time periods of several minutes. The mean firing rate was 4±6 Hz (mean±SD) for the incubated cells and 7±7 Hz for the fresh ganglion cells. Both cell populations show a similar histogram distribution with a few cells firing at high frequency. Incubated ganglion cells show a slightly reduced responsiveness as more cells are recorded with mean firing rates below 2 Hz.

The response of mammalian ganglion cells to a modulated visual stimulus consists of discrete firing events separated by periods of silence [19]. Thus, the simple firing rate of a ganglion cell is only a raw measure of responsiveness. The precision and reliability of firing events [20]–[22] were characterized by measuring the trial-to-trial jitter in response to intensity steps. The jitter has been found to be around 10 milliseconds in freshly prepared ganglion cells in the rabbit [20]. Because the mean jitter slightly depends on the stimulus contrast we compared the temporal jitter for 57 ganglion cells from incubated retinas with 59 ganglion cells from fresh retinas using the same stimulus protocol. The temporal jitter distributions were almost identical for the two populations (no statistical difference detected; p<0.05 ANOVA test). The mean population temporal jitter (Fig. 4 B, Figure S3) for incubated ganglion cells was 10.0±2.1 ms and 8.6±2.2 ms for fresh ganglion cells.

The spatial receptive fields were measured using a pseudo-random checkerboard stimulus modulated with a binary m-sequence (Fig. 4 C) [23], [24]. The mean diameter values were: 520±130 µm for incubated ganglion cells and 430±120 µm for fresh ganglion cells. This is within the variation expected for five samples that came from different retinal areas.

Although grossly normal in each of the three measures just described, the overall picture does hint at subtle differences between incubated and fresh retinas: incubated retinas have slightly lower firing rates, more variable timing of responses, and larger receptive fields (Figure 4). In aggregate these suggest that incubation does affect the performance of these cells, but none of them is by itself statistically significant and the effects are slight.

### Localization of fusion proteins of synaptic markers

**Figure 5:**
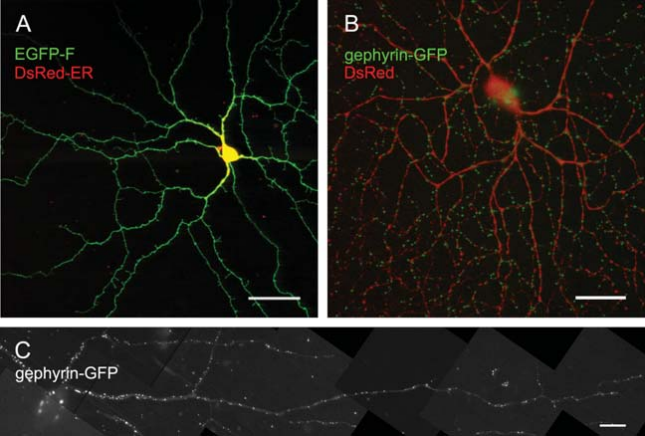
Expression of markers for subcellular structures in retinal ganglion cells. (**A**) Single confocal image plane of a large ON ganglion cell transfected with expression plasmids for EGFP-F (labeling the cell membrane) and DsRed2-ER (labeling the endoplasmic reticulum.) Scale bar, 50 µm. (**B**) Volume reconstruction of a confocal image stack of a large ganglion cell transfected with the cell-filling marker HcRed and a marker for postsynaptic densities of inhibitory synapses, gephyrin-GFP (green). Scale bar, 50 µm. (**C**) A single dendrite of an OFF alpha ganglion cell transfected with gephyrin-GFP. Note the high density of puncta particularly on the proximal dendrites. Scale bar, 20 µm.

We transfected retinal ganglion- and amacrine cells biolistically with plasmids expressing a variety of genes. They included GFP and its spectral variants; DsRed2, HcRed; EGFP-F (a cell membrane-bound variant of GFP); DsRed2-ER (coding for DsRed2 fused to endoplasmic reticulum targeting and retention sequences); fusion proteins of the synaptic markers PSD95-GFP, gephyrin-GFP, rapsyn-GFP, synaptophysin-CFP, VAChT-GFP, ankyrin-G-GFP; and siRNAs for the silencing of the GABA receptor γ chain. Figure 5 A shows the expression of EGFP-F in a ganglion cell.

**Figure 6:**
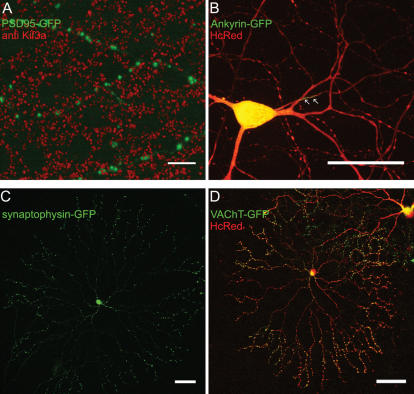
Pre- and postsynaptic markers and ankyrin-G are correctly localized in transfected retinal neurons. (**A**) High-power view of several dendrites of a retinal ganglion cell transfected with PSD95-GFP (green) and counterstained with an antibody against the synaptic ribbon component Kif3a (red). Most PSD95 puncta are in close proximity to one or more synaptic ribbons. Scale bar, 10 µm. (**B**) Ankyrin-G-MB-GFP (green) and HcRed (red) were transfected into a retinal ganglion cell. HcRed labels the whole extent of the cell, whereas Ankyrin-G-MB-GFP is found in the initial segment of the axon (arrows). Some Ankyrin-G-MB-GFP labeling is seen in the soma and the primary dendrites, the place of protein synthesis. Scale bar 50 µm. (**C**) Starburst amacrine cell transfected with an expression plasmid for synaptophsin-GFP. The known synaptic output sites of these cells, varicosities in the outer third of the dendritic tree are labeled with this marker. (**D**) Starburst amacrine cell transfected with expression plasmids for the cell-filling marker HcRed (red) and a fusion protein from the synaptic vesicle marker VAChT-GFP (green). Scale bar, 50 µm.

To evaluate the localization of fusion proteins in retinal neurons, we show here the expression of several GFP-tagged synaptic markers and of the sodium-channel organizing protein ankyrin-G. The postsynaptic glycine- and GABA-receptor organizing protein gephyrin is expressed as bright puncta on ganglion cell dendrites (Fig 5 B&C), consistent with its localization known from other studies [25]. We also expressed the postsynaptic density component PSD95 in retinal ganglion cells. As most of the excitatory input synapses to ganglion cells are from bipolar cells, most of the PSD95 puncta should be in close proximity to synaptic ribbons which in the inner retina are unique markers of bipolar cell vesicle release sites. We counterstained retinas after transfection with PSD95-GFP with an antibody against the synaptic ribbon component kinesin (Kif3a). As shown in figure 6 A, most of the PSD95 puncta are in the near vicinity of one or more synaptic ribbons. In contrast to PSD95 and gephyrin, which are localized in the dendrites, ankyrin-G expression is expected to lead to a signal in the initial segment of the ganglion cell's axon, the site of the densest concentration of sodium channels. Figure 6 B shows a ganglion cell with obvious labeling of the axon initial segment.

We also expressed the presynaptic markers synaptophysin and vesicular acetylcholine transporter (VAChT) as fusion proteins with GFP in starburst amacrine cells. Synaptophysin-GFP and VAChT-GFP labeled starburst varicosities restricted to the outer third of the dendritic arbor (Figure 6 F & G). This is in accord with the restricted localization of starburst synapses established by electron microscopy and Ca2+ imaging [26]–[28].

## Discussion

We sought to establish a system that is suitable to genetic manipulation, recording, imaging, and short-term pharmacological studies. In addition, it should be possible to process several samples in parallel, and the cultures should require as little maintenance as possible. The retina interphase culture meets these basic goals.

The main advantage of the retina culture system described here is that it allows expression of a wide variety of genes without generating transgenic animals or designing viral vectors. Particle-mediated transfection is rapid and flexible. Furthermore, combinations of genes can easily be transfected, by coating different plasmids simultaneously on the gold microcarriers – as we have shown here for combinations of a cell-filling marker protein and a targeted synaptic one. We have used rabbits, because the rabbit retina has been much studied, but the same method could be used for the culture of retinas of other species that are commonly used in vision research, like monkey or cat. Although the system also works for the retinas of mice, the experimenter is no longer is dependent on the mouse retina of whose thickness and small size makes it undesirable for retinal research.

Another advantage of the method reported here is that the experimenter has a range of choices of fluorophores for cell labeling. Furthermore, biolistic transfer is not restricted to nucleic acids. Cell-filling dyes –such as DiI, FITC-dextran-, or Ca^2+^ indicator dyes, can also be used [14], [29], [30]. The incubation requires minimal use of growth factors and neuroprotective agents. Finally, biolistically labeled ganglion cells on high-density electrode arrays may facilitate combined morphological and physiological studies – a frequent missing link in multielectrode array experiments aimed at the characterization of ganglion cell types.

There are several limitations to the technique. As an explant culture of the retina necessarily involves severing the optic nerve, the ganglion cells will eventually undergo retrograde degeneration. This may be an issue as certain biological effects (e.g. achieving a high level of inhibition by a siRNA construct) take several days to develop. The retina is separated from the retinal pigment epithelium during preparation, limiting the use of bright light for stimulation and risking interference with photoreceptor disk shedding.

At present no transfection technique – including gene gunning – is suitable as a population stain. The number of labeled cells can be influenced to some extent by the amount of microcarriers used, the interposition of filters, and the delivery pressure, but will rarely exceed 100 cells/mm^2^. This is advantageous for many experiments, as individual cells can be easily recognized morphologically, but it precludes studying the function of the whole network of cells of one type. For example, several recent papers describe the expression of fluorescent or non-fluorescent markers under the control of cell type-specific promoters [31]–[34]. This approach can be extended to eliminate all cells of a particular type from the retinal network, as has been demonstrated for starburst amacrine cells [35] and neuropeptide-Y positive amacrine cells [36]. For studies of this kind, the use of transgenic animals is indispensable.

The transfection technique used in our study is also biased toward transfection of cells near the surface. The frequency of cell types in a typical experiment is: ganglion cells>displaced amacrine cells>>amacrine cells>Mueller cells>bipolar cells>horizontal cells>photoreceptors (see Figures S4 & S5 for examples of a Mueller cell and a cone bipolar cell).

## Methods

### Isolation of rabbit retina

Light adapted New Zealand White Rabbits 5 to 12 weeks of age were used for transfection experiments. Handling and euthanasia of animals were done in accordance with the guidelines provided by the Subcommittee of Research Animal Care of the Massachusetts General Hospital. Rabbits were deeply anesthetized using 200 mg ketamine/40 mg xylazine and euthanized by an overdose of pentobarbital. The eyes were removed and immediately transferred to oxygenated Ames' medium (Sigma) for hemisection. The retinas were teased off the sclera using a fine brush. If the retina was to be used for recording of light responses, the rabbits were dark-adapted for one hour, the retina preparation was carried out under dim red light, and the cultures were protected from light until use.

### Interphase/Perfusion chamber for the explant culture of adult rabbit retina

Pieces of rabbit retina of ca 1 cm^2^ were placed ganglion cell side up on a 0.4 µm Millicell tissue culture insert (Millipore). The quality of the tissue after incubation depended on smooth attachment to the membrane; we found it indispensable to apply gentle suction to the tissue for ∼30 seconds during mounting. Filter stands (2 cm diameter, 1 cm high) were cut from the caps of 1 ml Monoject Tuberculin syringe jackets, so that the Millicell filter rested on four “stands” when it was placed into a 60×20 cell culture dish (Nunc). Approximately 25 ml Ames' medium (Sigma) containing 1% horse serum, 1% N2 supplement, and 100 U/ml penicillin, 100 U/ml streptomycin, 0.3 mg/ml L-glutamine (Invitrogen) were added to the dish, so that the retina was in contact with the medium via the Millicell filter over the photoreceptor side, and with the incubator atmosphere (5% CO_2_, 35°C, humidified) over the ganglion cell side. All further manipulations, including gene gunning, were carried out with the retina attached to the Millicell filter. The medium was exchanged daily.

### Cell viability assay

Cell viability was assessed using a Vybrant apoptosis assay kit (Invitrogen). Rabbit retinas directly after preparation or after 2, 4, or 6 days of culture were incubated with 100 nM YO-PRO-1 and 100 nM propidium iodide for 1 h. Dead cells (labeled in red), apoptotic cells (labeled green), and healthy cells (labeled dimly green) were counted in four separate fields per retina.

### Biolistic transfer of plasmids or dyes

A Helios gene gun system (BioRad) was used for particle-mediated transfer of DNA or dye molecules to retinal neurons. The expression plasmids for EGFP, ECFP, HcRed, DsRed2-ER, and EGFP-F were obtained from Clontech. The expression plasmid for the PSD95-GFP fusion protein was a gift of Dr. Morgan Sheng (Picover Center for Memory and Learning, Cambridge, MA). The expression plasmid for gephyrin-GFP [37] was kindly provided by Dr. Jochen Meier (Charité, Berlin, Germany). The Ankyrin-G-MB-GFP plasmid [38] was a gift from Dr. Edward Cooper (University of Pennsylvania [39]). The expression plasmid for synaptophysin-CFP was obtained by inserting a PCR fragment corresponding to the coding region of the gene into the pECFP-N1 vector (Clontech). Plasmid-coated gold microcarriers (1.6 µm gold) were prepared as described in the manufacturer's protocol. The gene gun was loaded with gold microcarriers coated with the appropriate plasmids (usually at 1 µg DNA per mg gold), held 2 cm above the tissue, and the microcarriers were propelled into the retina at a delivery pressure of 110 psi. The retina was then washed in Ames' medium and reinserted into the dish.

### Immunohistochemistry and image acquisition

Two to six days after transfection the retinas were harvested and immunostained. Antibodies against ChAT (goat, Chemicon) and Kif3a (kinesin, mouse monoclonal, Covance) were used. Secondary antibodies obtained from Jackson Immunoresearch. In some cases the retinas were counterstained with the nuclear dye TOPRO-3 (Invitrogen).

Individual cells were imaged on a confocal microscope (Radiance, BioRad) using water immersion objectives (25x/0.8 Plan Apochromat, 40x/1.2 C-Neofluar, or 63x/1.4 C-Neofluar, all from Carl Zeiss Microimaging). Through-focus series of images were taken of labeled cells in the ganglion cell layer. The images were median filtered and collapsed into a maximum intensity projection by user-written routines in Matlab (The Mathworks), or volume reconstructed (VolView 2, Kitware). The resulting images were adjusted for brightness and contrast in Photoshop CS (Adobe).

### Recording from retinas after incubation

Patch pipettes (10–15 MΩ resistance) were pulled from Pyrex tubing on a micropipette puller (P-97; Sutter Instrument). The pipette solution consisted of: (in mM): 87 K-gluconate, 7 KCl, 5 HEPES, 0.4 CaCl2, 0.4 MgCl2, 5 EGTA and 2 ATP-Na_2_ (pH adjusted to 7.4 with KOH). The piece of retina was placed in a recording chamber, ganglion cell layer up, and continuously perfused at a rate of 2 ml/min with oxygenated Ames' solution at 32–35°C. Cells were viewed using a microscope (Axioscope, Carl Zeiss Inc.) equipped with infra-red differential interference contrast optics. The recording pipette was connected to the input stage of a patch-clamp amplifier Axopatch 200B; Axon Instruments, Foster City, CA); and signals were sampled at 10 kHz with DigiData 1322A interface-type and pCLAMP8 software (Axon Instruments). Liquid junction potential was corrected after recordings (Vm = Vrecord–17 mV). Photo-stimuli were generated on a calibrated CRT monitor (Dell-P780, SONY) using Visionworks (Vision Research Graphics) and reflected upward by a mirror positioned underneath the preparation. A microscope objective (Olympus Optical; 20x, N.A. 0.4) focused the stimulus on the retina. Data were analyzed with user-written routines in Igor Pro (Wavemetrics).

Multielectrode array recording of rabbit retina was described in detail elsewhere [40]. Recordings of ganglion cell activity was performed after 2 or 3 days of incubation on an array of 60 electrodes spaced 100 µm apart (Multichannel Systems). The extracellular waveforms were recorded when their amplitude exceeded a threshold set at 2.5 SD above mean noise level, and stored at 33 µs time resolution. A supervised neural network algorithm (Spike Sorter IV, Cyberkinetics) separated the waveforms and stored them as cell-specific time-stamped spike trains. Spike train analysis was performed using user written routines in MATLAB. Photo-stimuli were generated as described above. The receptive field was mapped using a 16×16 pseudo-random flickering checkerboard (75 µm checkersize); the luminance of each square was independently modulated by a binary m-sequence [24]. Temporal precision was checked with a white noise full field flicker stimulus (temporal flat power spectrum in the 1–30 Hz range). The stimulus was 30 second long and repeated between 20 and 30 times.

## Supporting Information

Figure S1 Vertical sections through rabbit retina incubated for four days. Panel A, single confocal image of a retina stained with an antibody against calbindin, staining cells in the inner nuclear and ganglion cell layers, and horizontal cells. Panel B, single confocal image of a retina stained with an antibody against the rod bipolar cell marker PKCα. Scale bar for both panels, 50 µm.
(1.23 MB TIF)

Figure S2 Directionally selective ganglion cell and starburst amacrine cell. Panel A, A directionally selective ganglion cell and an ON starburst cell (both in green) were found close to each other in a piece of transfected retina. The retina was counterstained with an antibody against ChAT (red) to reveal the starburst amacrine cells. DS ganglion cells are bistratified, the focus in panel A is on the OFF layer. Panel B, the same DS cell, focus on the ON layer. Scale bar for A & B, 50 µm. Panel C, vertical slice through a rabbit retina at high magnification. The dendrites of a DS ganglion cell are shown in green. The retina was counterstained with an antibody against ChAT (red). Note the two dendritic strata of the DS cell, both co-stratifying closely with the starburst amacrine cells' dendrites. The cell body belongs to an ON starburst cell. Scale bar, 20 µm.
(1.87 MB TIF)

Figure S3 Calculation of temporal response jitter to the presentation of repeated full-field flicker. Panel A, responses to 25 repeats of the same stimulus are shown as rasters in which each row represents a 5 sec segment of the neural response, and each dot represents the time of occurrence of a spike. Panel B, a narrow peak in the post-stimulus time histogram in the marked area in panel A, expanded and binned at 1 msec resolution. The PSTH histogram can be approximated by a Gaussian, the width thereof represents the temporal jitter. Note that in the histogram multiple spikes of one firing event are averaged.
(1.08 MB TIF)

Figure S4 High-resolution image of a Mueller cell. Panel A, B, and C, different views of a volume reconstruction of a Mueller cell from 353 individual image planes. Views from the scleral side (panel A), the vertical plane (panel B), and the vitreal side (panel C) are shown. Scale bar, 20 µm. The 12 images mounted below correspond to individual image planes at the positions indicated in B.
(5.43 MB TIF)

Figure S5 High-resolution image of an ON cone bipolar cell. Panel A & B, two different side views of a volume reconstruction of a cone bipolar cell from 153 individual image planes. Panel C, dendrites and cell body seen from the scleral side, the axon has been cropped out. Panel D, axonal termination of the same bipolar cell. The extent of the axonal termination is indicated by a white polygon. The thicker processes seen in the picture belong to a ganglion cell close by that was also labeled. Scale bars for all panels, 10 µm.
(6.31 MB TIF)
