# Supplementary figures and images for: Organotypic Culture of Physiologically Functional Adult Mammalian Retinas
Source: PLoS One. 2007 Feb 21;2(2):e221. doi: 10.1371/journal.pone.0000221 (PMC1794165; doi:10.1371/journal.pone.0000221)

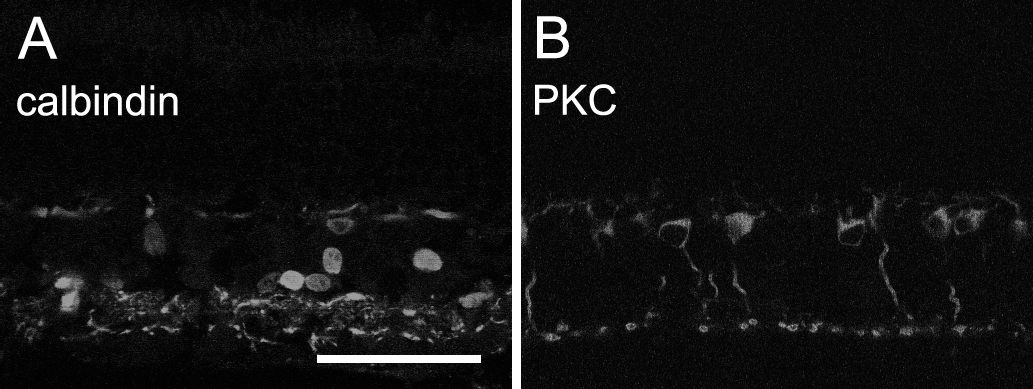

Supplement: Figure S1 — Vertical sections through rabbit retina incubated for four days. Panel A, single confocal image of a retina stained with an antibody against calbindin, staining cells in the inner nuclear and ganglion cell layers, and horizontal cells. Panel B, single confocal image of a retina stained with an antibody against the rod bipolar cell marker PKCα. Scale bar for both panels, 50 µm. (1.23 MB TIF) [file pone.0000221.s001.tif]

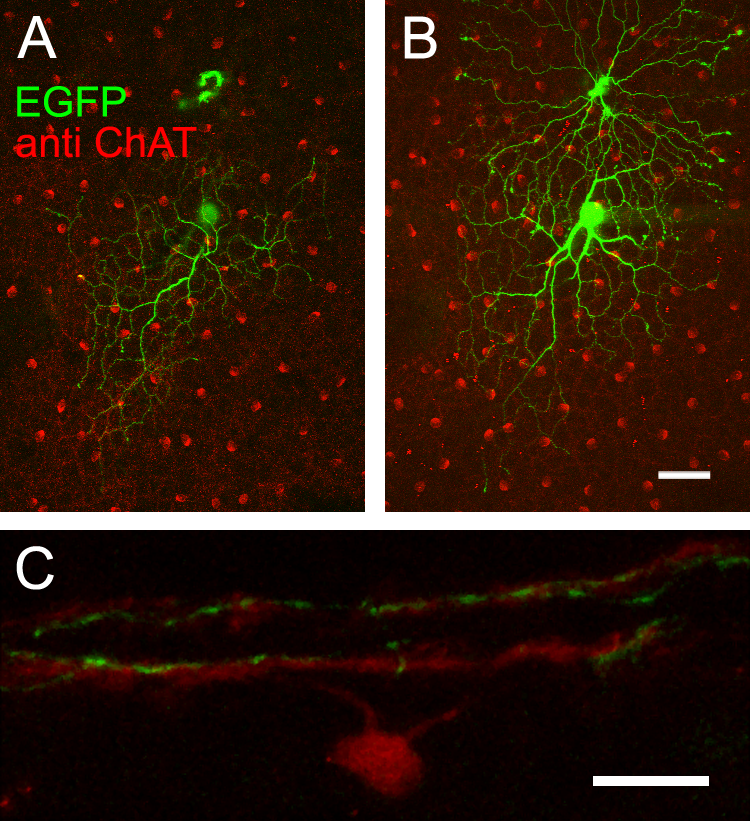

Supplement: Figure S2 — Directionally selective ganglion cell and starburst amacrine cell. Panel A, A directionally selective ganglion cell and an ON starburst cell (both in green) were found close to each other in a piece of transfected retina. The retina was counterstained with an antibody against ChAT (red) to reveal the starburst amacrine cells. DS ganglion cells are bistratified, the focus in panel A is on the OFF layer. Panel B, the same DS cell, focus on the ON layer. Scale bar for A & B, 50 µm. Panel C, vertical slice through a rabbit retina at high magnification. The dendrites of a DS ganglion cell are shown in green. The retina was counterstained with an antibody against ChAT (red). Note the two dendritic strata of the DS cell, both co-stratifying closely with the starburst amacrine cells' dendrites. The cell body belongs to an ON starburst cell. Scale bar, 20 µm. (1.87 MB TIF) [file pone.0000221.s002.tif]

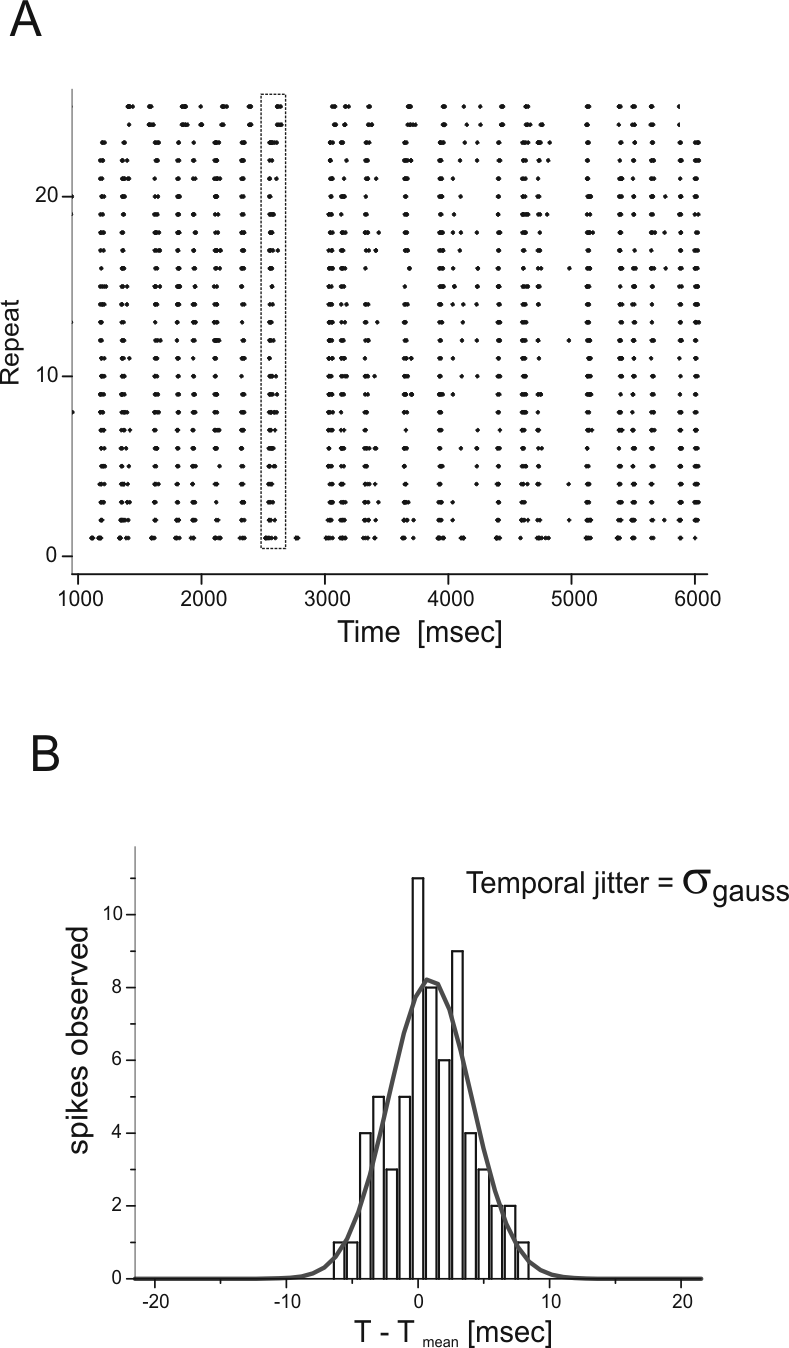

Supplement: Figure S3 — Calculation of temporal response jitter to the presentation of repeated full-field flicker. Panel A, responses to 25 repeats of the same stimulus are shown as rasters in which each row represents a 5 sec segment of the neural response, and each dot represents the time of occurrence of a spike. Panel B, a narrow peak in the post-stimulus time histogram in the marked area in panel A, expanded and binned at 1 msec resolution. The PSTH histogram can be approximated by a Gaussian, the width thereof represents the temporal jitter. Note that in the histogram multiple spikes of one firing event are averaged. (1.08 MB TIF) [file pone.0000221.s003.tif]

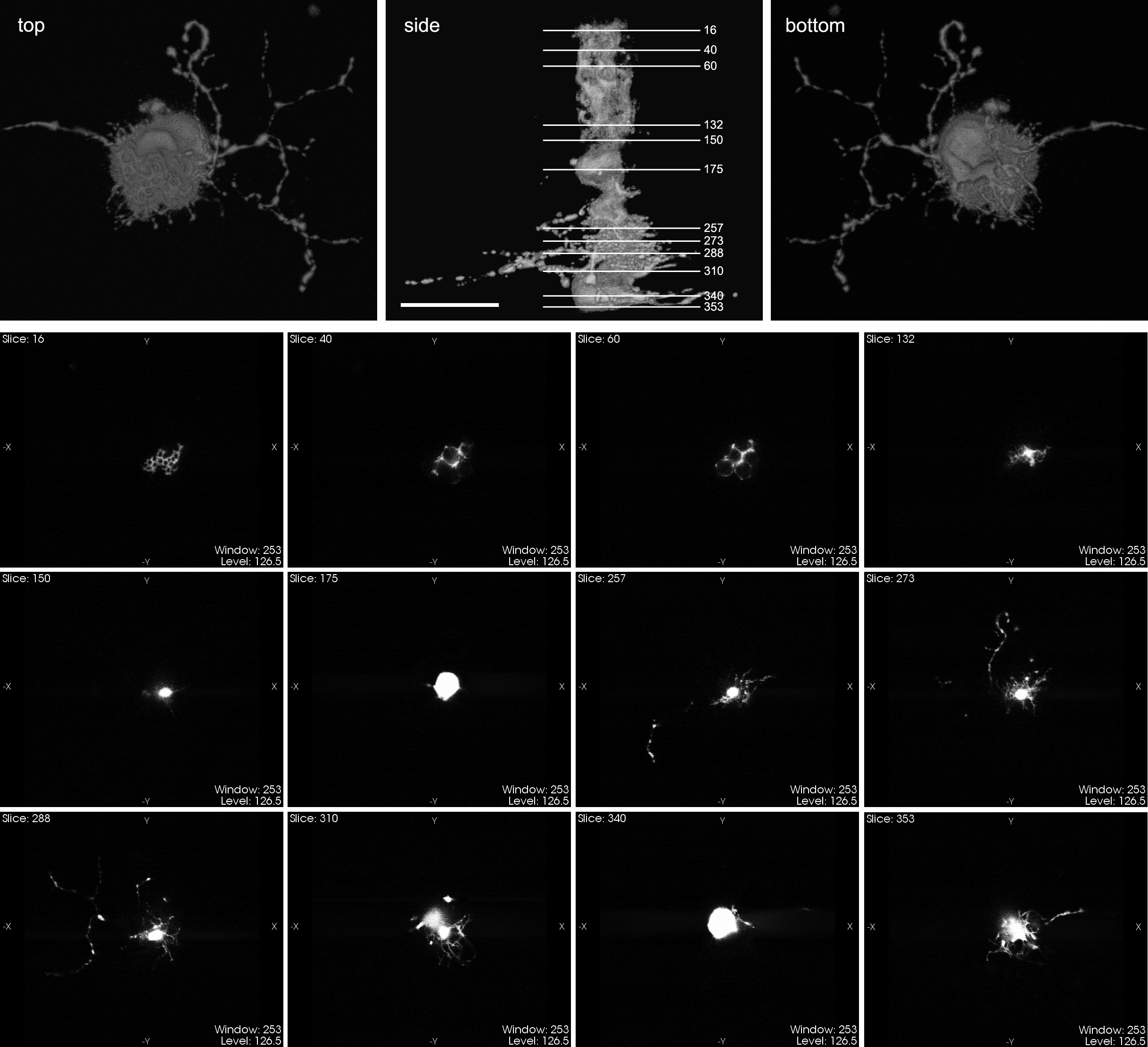

Supplement: Figure S4 — High-resolution image of a Mueller cell. Panel A, B, and C, different views of a volume reconstruction of a Mueller cell from 353 individual image planes. Views from the scleral side (panel A), the vertical plane (panel B), and the vitreal side (panel C) are shown. Scale bar, 20 µm. The 12 images mounted below correspond to individual image planes at the positions indicated in B. (5.43 MB TIF) [file pone.0000221.s004.tif]

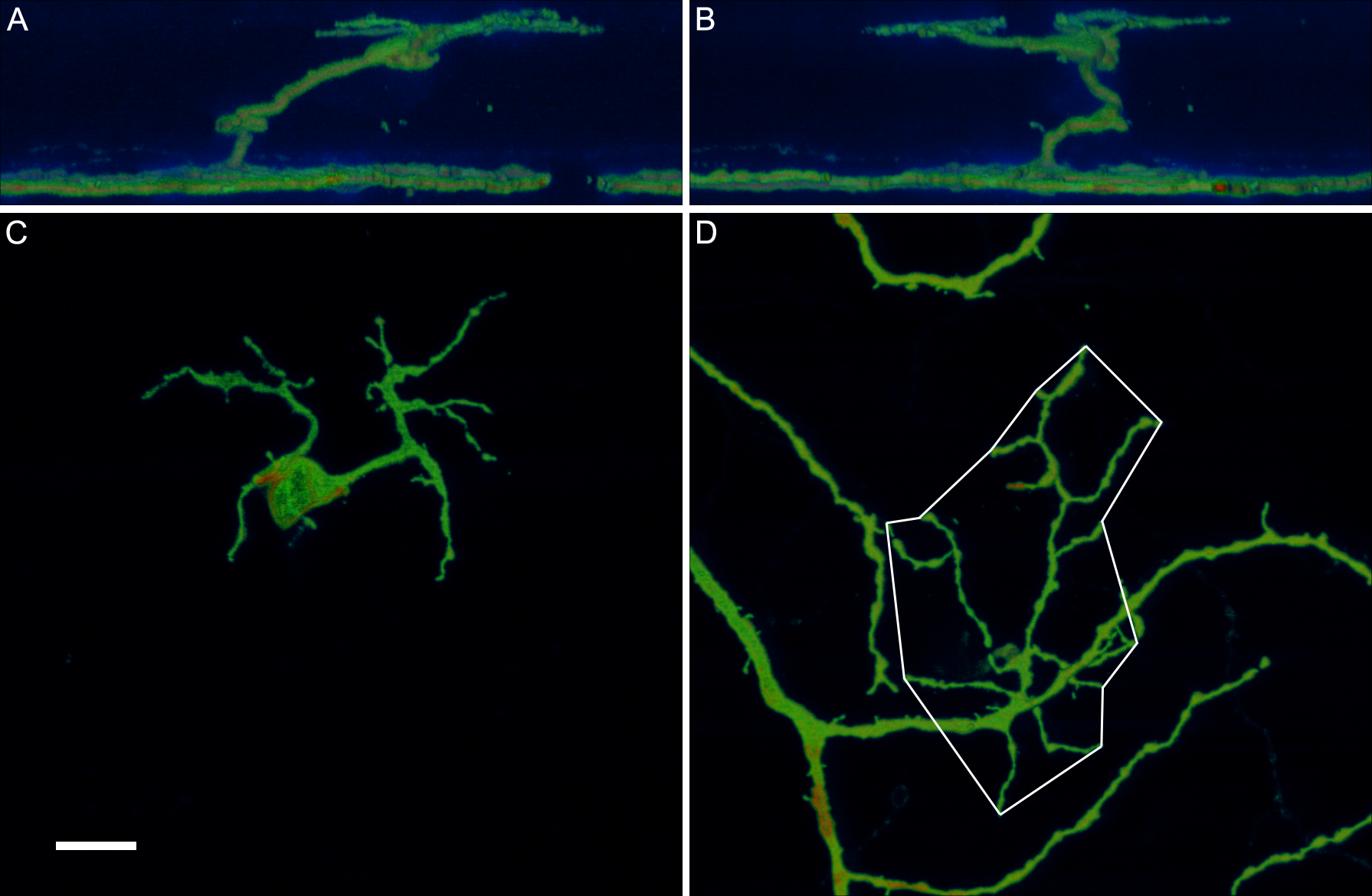

Supplement: Figure S5 — High-resolution image of an ON cone bipolar cell. Panel A & B, two different side views of a volume reconstruction of a cone bipolar cell from 153 individual image planes. Panel C, dendrites and cell body seen from the scleral side, the axon has been cropped out. Panel D, axonal termination of the same bipolar cell. The extent of the axonal termination is indicated by a white polygon. The thicker processes seen in the picture belong to a ganglion cell close by that was also labeled. Scale bars for all panels, 10 µm. (6.31 MB TIF) [file pone.0000221.s005.tif]
